# Centrally inserted central catheter placement using a novel, handheld, image-guided, robotic device: Results of initial feasibility trial in patients

**DOI:** 10.1177/11297298241273637

**Published:** 2025-02-10

**Authors:** James P Herlihy, William E Cohn, Adrian Ebner

**Affiliations:** 1 Baylor College of Medicine, Houston, TX, USA; 2 Texas Heart Institute, Houston, TX, USA; 3 Universidad Nacional de Asuncion, Asuncion, Paraguay

**Keywords:** Techniques and procedures, new devices, intensive care, catheters, dialysis access, interventional radiology

## Abstract

**Background:**

Central venous access devices (CVADs) are an essential and widely used tool for the treatment of the critically ill, patients undergoing major surgery, and for many patients requiring hemodialysis. Automation of centrally inserted central catheters (CICCs) could potentially make CVAD placement safer, more effective, and more accessible. A new device that uses ultrasound image-guided, robotic needle placement, in addition to traditional Seldinger technique, to place a CICC is described.

**Objective:**

The device was used in a small, first-in-human, trial for placing non-tunneled hemodialysis catheters (NTHDCs), in order to determine feasibility of clinical use.

**Methods:**

Consecutive patients requiring a NTHDC, at one institution, over a 48 h period, were recruited to consent to placing the catheter by the device. Observations of safety, efficacy, and efficiency of the procedure were recorded.

**Results:**

There were 19 catheter placement attempts in 17 patients. All placements were successful (100%). The first placement attempt was successful in 16/19 catheterizations (84%). Two catheterizations required two attempts and one required three attempts. There were no complications for any catheterization. The device provided rapid access to the target central vein and required relatively little training time for operators.

**Conclusions:**

The study demonstrates the feasibility for clinical application of a novel central venous access robotic device.

## Introduction

Central venous access devices (CVADs), and particularly centrally inserted central catheters (CICCs) are a fundamental tool for the practice of critical care medicine, trauma resuscitation, major surgeries, delivery of parenteral nutrition and, often, for providing hemodialysis. Not surprisingly, therefore, at least 7 million CICCs are placed in the United States per year.^1,2^ However, there is a substantial level of training, and experience required to place these devices safely and effectively.^3^ Skilled resources for CICC placement are often not readily available in community care settings. Even with experienced providers placing these lines, failure of placement has been reported to be surprisingly high, at 9.2% in a recent multicenter prospective trial looking at CICC complications.^4^ The largest prospective study of CICC placement outcomes demonstrated that even with near 100% use of ultrasound (US) guidance, 15% of placement attempts involved multiple needle stick attempts to establish venous access.^5^ Often, CICCs are placed in emergent situations where rapid central venous access is key to favorable outcomes.^6,7^ A recent emergency department study showed only a 62% success rate by experienced operators for the initial attempt at CICC placement, and the procedural time was, on average, greater than 30 min.^7^

CICC insertion is also associated with significant complications, to include pneumothorax and bleeding. Complication rates depend upon the anatomic location of catheter placement, experience of the proceduralist, and certain patient characteristics such as body habitus.^8^ Using best current practices, the overall complication rate for CICC placement is approximately 7%.^5^

Automation of CICC placement has held promise to make central catheterization safer and more reliable, as well as easier, faster, and more accessible.^9,10^ A number of technical barriers have, however, prevented the emergence of a practical, automated CICC placement device.^11^ We describe here a device designed to overcome these barriers and provide a practical advance in bedside placement of CICCs. After extensive testing in a large animal model^12^ the device was studied for safety, efficacy, and efficiency of use in patients, via a limited observational trial, to determine feasibility of using the device clinically to place non-tunneled hemodialysis catheters (NTHDCs).

## Methods and patients

### Study device

**Figure 1.**
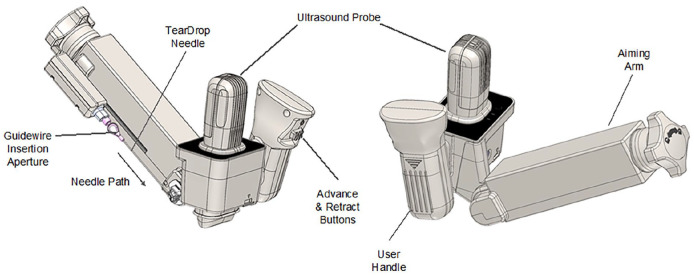
CERTA Access Device. The ultrasound probe is a standard linear ultrasound for imaging the target central vein. The User Handle is designed for one-handed manipulation of the device for positioning the ultrasound probe. The Aiming Arm holds the Teardrop Needle, the tip of which is automatically advanced into the target vein (the targeting mechanism is shown in Figure 2) by a linear actuator in the arm while being vibrated. The Teardrop Needle (shown in more detail in Figure 3) is used for both vessel access and for placement of a guidewire, though the unique basin design, into the vessel. The Aiming Arm is locked into position once the target vessel is selected and before advancement of the needle. The device mechanics ensure the needle does not advance beyond the selected target. The device is battery powered and wireless, and the handle includes push button control of needle advancement and retraction.

**Figure 2.**
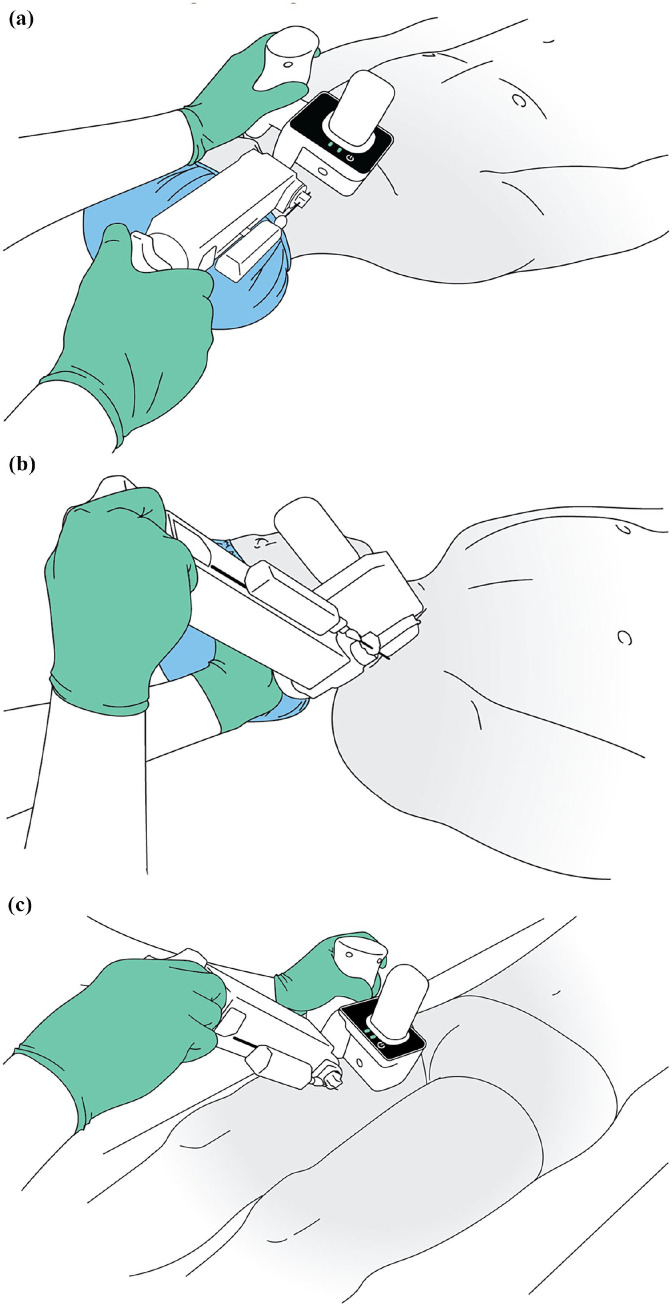
Positioning of CERTA Access Device for placing CICCs: (a) placement for right internal jugular vein access, (b) placement for right subclavian vein access, and (c) placement for right femoral vein access.

The CERTA Access System (or CERTA, for short) from Obvius Robotics (Sunrise, Florida, USA), is a handheld, electromechanical device that enables targeting of a central vein for needle placement with the use of an integrated ultrasound (US) probe, and automatic advancement of an access needle into the target vessel. CERTA consists of the CERTA Access Device (Figure 1), the CERTA Tablet and image viewer application (shown in Figure 2), and the CERTA Sterile Drape Kit.

### Device operation

In brief, the device allows the operator to visualize and target a central vein, with the integrated US, from traditional patient surface approaches for placing CICCs (Figure 2). Once a target in the central vein is selected, an access needle, similar to needles used in a manual central venous access, is automatically advanced into the vein. The needle access allows a guide wire to be placed in the vein, and a catheter to be placed over the wire and into the vein, via Seldinger technique.

### Step by step approach

**Figure 3.**
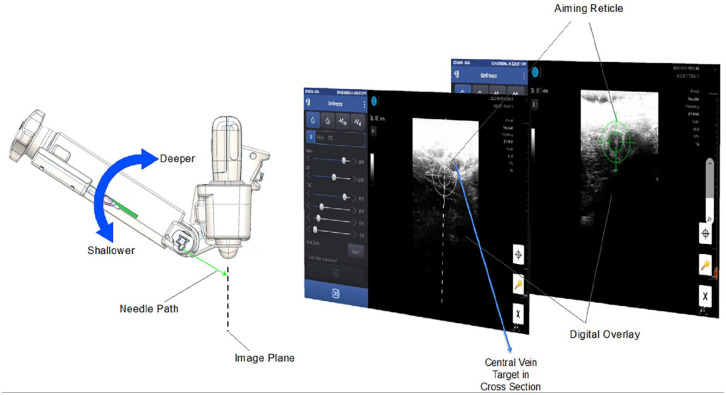
Targeting central vein. The target central vein is viewed in short axis on the CERTA Tablet, for the procedure. An aiming reticle, initially white in color, is used to guide the “Aiming Arm” into correct position, demonstrated by the reticle turning green, to deploy the Teardrop Access needle.

**Figure 4.**
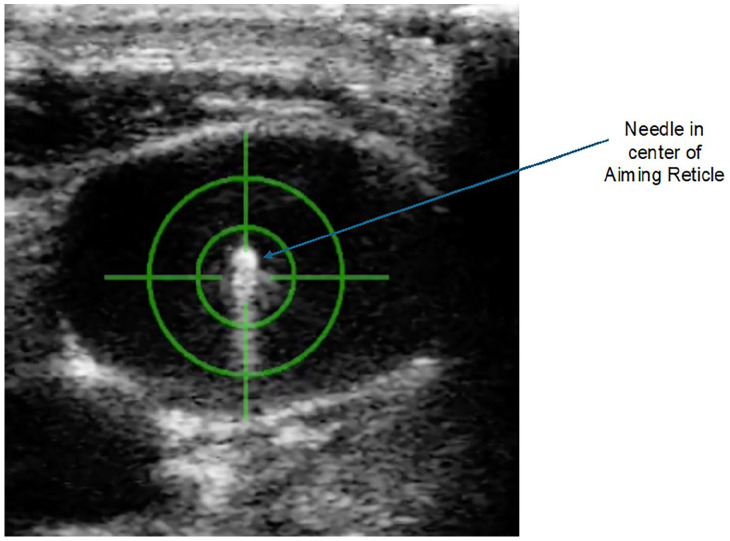
Needle confirmation by US. The needle tip can be seen in the central vein, one element of confirmation for vessel access.

**Figure 5.**
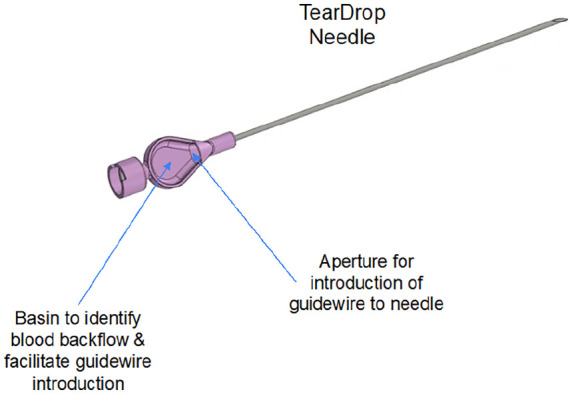
Teardrop Needle. TearDrop Needle showing basin for blood backflow and guidewire introduction.

The target vessel for a CICC is typically viewed in long and short axis prior to the procedure, for suitability of catheter placement, via the US probe integrated into the device. A “short axis, out of plane technique” is employed for needle placement. Step 1 of the procedure is to select, by US, the cross sectional area of the vein where the central venous access needle tip is to be placed. Step 2 of the procedure is to position the central venous access needle, held by the Aiming Arm of the device, so that, when advanced, the tip will go the vessel center (Figure 3). The Aiming Arm is a component of the CERTA Access Device, along with the integrated US module and device handle. The Aiming Arm is moved up and down manually, but in a fixed tract along a Euclidean arch, in a plane perpendicular to the cross-sectional US image. The movement along the arch positions the needle to enter the skin at different angles, and for advancement to different depths. An aiming reticle, with cross hairs, works in registration with the Aiming Arm movement, to overlay a target for the needle tip onto the US image. Once the target is selected by the user, the aiming arm is locked into position. Step 3 is advancement of the needle automatically by the push of a button on the device handle. The next step of the procedure is to confirm that the needle tip is properly positioned in the vessel center by visual confirmation from the US image (Figure 4) as well as flow of venous blood into the basin of the TearDrop Needle (Figure 5). The spoon shaped basin of the TearDrop Needle was specifically designed for the CERTA Access System, and allows for easy visualization of back flow blood (a standard for confirming venous entry by traditional CICC manual placement technique), while also facilitating the next step of the procedure, introduction of the guidewire into the vein, to be conducted under US guidance.

The device handle is designed to be held by the left hand and the guide wire placed by the right hand. Both right-and left-handed operators were incorporated into user testing and both found the device facile to use.

It is important to note that the device applies axial micro vibrations to the needle as it advances. This vibration reduces the force of needle penetration and the consequent tissue deformation that occurs with manual needle insertion. The reduction of tissue deformation, in turn, reduces the movement of the venous target during needle insertion, a key to accurate needle placement. Furthermore, the needle cannot advance beyond the target selected by the user with the Aiming Reticle due to the device’s control system. This reduces the risk of the operator advancing the needle too deep, in turn offering the potential for reduced insertion complications.

Of note, the subclavian (SC) vein approach was infraclavicular and proceeded by identifying the axillary vein first and tracing it medially to the SC vein. Once a target location for access was established under US, the “short axis out of plane” technique was employed for SC vessel access in the same manner as for the internal jugular (IJ) and femoral vein approaches.

### Study protocol

The study location was Sanatorio Italiano, in Asunción, Paraguay, during the month of May 2023. The study was conducted according to ISO 14155:2020 and approved by El Comité de Ética de la Investigación del Instituto Paraguayo de Estudios Sociales (Clinical Research Protocol, CIP ARC-3003). In brief, the study population consisted of patients in need of a non-tunneled hemodialysis catheter (NTHDC). The primary intervention was the use of CERTA, along with the Seldinger technique, to place the catheter in a central vein. The procedure operators were three local staff interventional cardiologists, experienced in placement of CICCs of all types, as well as one of the authors (WEC), a cardiovascular surgeon also experienced in the placement of CICCs. The training to competence in device usage consisted of a didactic introduction to the principles of the device operation by a company representative, and hands on use of the device to place catheters in a standard commercial mannequin model for training CICC placement. Training took approximately 1 h. Measures of safety, efficacy, and efficiency were obtained for the procedure by an independent research coordinator.

Specific study candidate criteria:

### Inclusion criteria for the study

1. Age >18 and <80 years at the time of consent.
2. Urgent or emergent indication for hemodialysis, for new onset renal failure, or Continual Renal Replacement Therapy (CRRT), where no adequate dialysis access site was available.
a. Central venous access indicated for the placement of a non-tunneled hemodialysis catheter (NTHC).
3. Patients able to sign a study Informed Consent Form.

### Exclusion criteria

1. Coagulopathy.
2. Evidence of localized infection at potential catheter insertion site(s).
3. Previous coronary interventional procedure of any kind within 30 days of the procedure.
4. Venous obstruction proximal or distal to the site of the catheter insertion.
5. Inability to comply with the study protocol, for whatever reason.
6. Current participation in any other investigational drug or device study.

Consecutive candidates, over a 48-h period, were offered the opportunity to participate in the study via a detailed study-specific informed consent, that described the device and its operation in lay language. The target vessels for the procedure were preferentially, in order, the right IJ (RIJ) vein, the left IJ (LIJ) vein, the left SC (LSC) vein, the right SC (RSC) vein, and finally femoral veins, as last resort. This deviation from the Kidney Disease Outcomes Quality Initiative (KDOQI) guidelines^13^ was due to institutional concern for Central Line Associated Blood Stream Infection (CLABSI) from catheters in the femoral position,^14^ in this particular cohort of patients. The specific site per patient was determined by a scout US with the CERTA device showing an accessible vessel, with no obvious distal obstruction, of greater than 9 mm in diameter. After consent the target vessel area was prepped and draped in standard, sterile fashion to include the use of a transparent sterile drape over the reusable CERTA Access Device. Local lidocaine was administered. A NTHDC was then attempted using CERTA via the technique described above in Device operation. After the procedure, the CERTA Access Device, was cleaned and disinfected with a germicidal wipe containing isopropyl alcohol.

### Data collection

#### Safety data

The CVC site accessed by CERTA was observed for 12 h following the procedure, by the proceduralist. Data collection sheets, maintained by the independent research coordinator, recorded by the any of the following complications:

- Arterial puncture
- Major bleeding requiring a local surgical intervention (sutures, etc.), or volume resuscitation.
- Local hematoma
- Pneumothorax
- Hemothorax
- Venous air embolism
- Local trauma to nonvascular structures

#### Efficacy data

- CERTA access attempts that resulted in placement of CVC (success), or failure to place, were recorded.

#### Efficiency data

- Access time (defined as time from the beginning of ultrasound visualization of the target vessel after sterile set up, to guidewire introduction) for each attempt at central venous cannulation by CERTA was recorded.

## Results

Seventeen patients had 19 CVC placement attempts with CERTA. Eighteen catheters were NTHDCs. One catheter placement was of a multi-lumen CICC placed in the left femoral position emergently, as the patient suffered a cardiac arrest while being prepped for NTHDC placement and required immediate resuscitation. There were 10 males and seven females. Ages range from 27 to 76 years, with a mean of 53 years. BMI ranged from 17.8 to 34.7 kg/m^2^ with a mean of 25.1 kg/m^2^. There were several obese patients with an endomorphic body habitus.

### Complications

There were no complications recorded for any uses of CERTA.

### Efficacy

Catheterizations of all 17 patients, and 19 catheterization attempts, were successful. The location of CICCs and the number of attempts to gain vessel access through CERTA, are presented in Table 1. First attempt success occurred in 16 of 19 cases, yielding a success rate of 84.2%. Two catheterizations required two attempts. In one, the patient moved slightly during the procedure, and in the other, the operator’s positioning of the CERTA Access Device moved slightly. One patient required three attempts. That patient was assessed as being volume depleted and required greater Trendelenburg positioning, before access was gained. One patient suffered a cardiac arrest while sterile prepping for NTHDC. That patient had a multi-lumen CICC placed in the left femoral position emergently via CERTA with first attempt success. The patient was effectively resuscitated and subsequently had the dialysis catheter after access was achieved with CERTA.

**Table 1.** CVC procedures by anatomic site using the CERTA access system.

Site	Procedures attempted	Procedures successful	Number of insertion attempts/pt		
			1	2	3
R IJ	12	12	9	2	1
L IJ	2	2	2	0	0
R SC	1	1	1	0	0
L SC	3	3	3	0	0
R fem	0	0	0	0	0
L fem	1	1	1	0	0
Total	19	19	16	2	1

R IJ: right internal jugular vein; L IJ: left internal jugular vein; R SC: right subclavian vein; L IJ: left subclavian vein; R fem: right femoral vein; L fem: left femoral vein.

### Efficiency

The mean time to gain guidewire access with CERTA was 3 min and 21 s. The minimum time was 56 s and the maximum 12 min and 58 s. The longest case involved three access attempts. See above.

## Discussion

Seventeen patients had 19 CICCs placed with CERTA without a complication and with a 100% success rate. First attempt success rate was 84.2%. Though numbers are small in this study, these outcomes compare favorably to complication and placement success rates reported in recent studies.^4,5^ Once patients were prepped, the average time to venous access by CERTA was 3 min and 21 s. Though time to venous access by standard CVC insertion technique, as a comparison, was not measured in this study, the speed of CERTA enabled CICC placement compares well to our experience, and what little is published in the literature.^7^

Three catheter placements required more than one attempt. Two of the initial failures were due to patient movement and inadvertent operator movement of the device during needle advancement. These movements obviously decouple the target from the needle trajectory. However, neither resulted in harm. Unexpected patient movement is a potential cause of procedure failure using traditional manual insertion technique as well. The needle advancement in the CERTA Access Device occurs over 3 s, but even so, patients can move in that short interval. Manual insertion of a needle into a central vein can easily be misdirected by poor visualization of the needle itself, poor path planning, and poorly executed manipulation of the needle advancement. Similarly, access needle advancement can be misdirected by an operator inadvertently moving the skin surface position of the CERTA Access Device, before or during needle deployment. One of the advantages of CERTA is that the needle will not advance past the original target distance. Many complications of CICC placement are due to over advancement of the needle into vulnerable structures.

The major limitation of this study is that its small sample size, single center locus, and limited group of investigators, does not allow definitive conclusions for the relative safety, efficacy, or even efficiency of CERTA placement of CICCs to current technique. The results do, however, encourage and demonstrate the feasibility of a larger study designed and powered to address these questions.^15^ How the device would perform in different clinical conditions, at different anatomical sites, in patients with different body types, using different CICCs, with different skill level providers, are also important questions that our limited trial cannot answer at this time.

Several innovative aspects of CERTA design, as well as some constraints of this design, are worth highlighting.

Current CVC placement technique requires a provider to identify a target vein by ultrasound, visualize a needle path to that target, and then manually guide the needle along that path, while tracking its progress via US. A great deal of experience and “muscle memory” is required for success with this technique. As well, it is often the case that the needle tip cannot be clearly visualized by US throughout its advancement. CERTA, on the other hand, allows a one-time targeting of the vessel and automates advancement of the needle tip to that specific location via the “short axis out of plane” technique.

The passage of a needle through tissue displaces deformable soft tissues within the clinical path. Specifically, when a standard needle is advanced through skin and subcutaneous tissue toward a target vein, that vein is often pushed into a different position.^11^ This makes tract planning for needle advancement less reliable and subject to both operator and patient variability. Additionally, central venous wall deformation by an access needle presents a challenge. Attempts to manually push the needle through the wall often result in compression of the vein, rather than entry into the vein. Occasionally, a needle will suddenly “pop” though the front and back wall of a compressed vein and hit a deep structure, such as an artery or lung. Tissue deformation, and consequent target vein movement, has been perhaps the biggest challenge to automating central venous access.^11^ Among potential solutions to this challenge has been the development of vibrating needles, which reduce the force of insertion and consequent tissue deformation.^16,17^ The proprietary needle vibration system incorporated into CERTA reduces insertion force, mitigating tissue deformation, including that of vein walls, to allow the access needle to smoothly traverse tissue into the central vein target. Additionally, the break stop function of CERTA prevents the needle tip from advancing beyond the distance of the venous target and creating complications.

Automation of guidewire placement was investigated in previous research efforts by other parties in the pursuit of an automated solution to CICC. However, it proved difficult to detect downstream vessel obstruction by advancement resistance of the standard soft, flexible J-tipped guidewires. In our experience, tactile feedback with manual advancement of the wire has proven to be more useful.

The design of the custom needle and associated basin (TearDrop Needle) is an innovation that allows for rapid one-handed guidewire insertion once the vessel is entered. The TearDrop Needle does not require detachment from a syringe (current process) before the catheter guidewire is advanced, and allows for guidewire introduction while maintaining US visualization.

## Conclusion

Automation of central venous access can potentially enable safer, more reliable, and more efficient placement of CVCs, with less training and experience. This report on the first human use of CERTA for placement of CVCs demonstrates the clinical feasibility of such an approach.
